# Supplementary material for: Venomix: a simple bioinformatic pipeline for identifying and characterizing toxin gene candidates from transcriptomic data
Source: PeerJ. 2018 Jul 31;6:e5361. doi: 10.7717/peerj.5361 (PMC6074769; doi:10.7717/peerj.5361)
Supplement: Supplemental Information 6 [file peerj-06-5361-s006.gz › FinalOutput_1E-6/Zinc_metalloproteinase-disintegrin-like_VAP2B_272/finaltree.pdf]

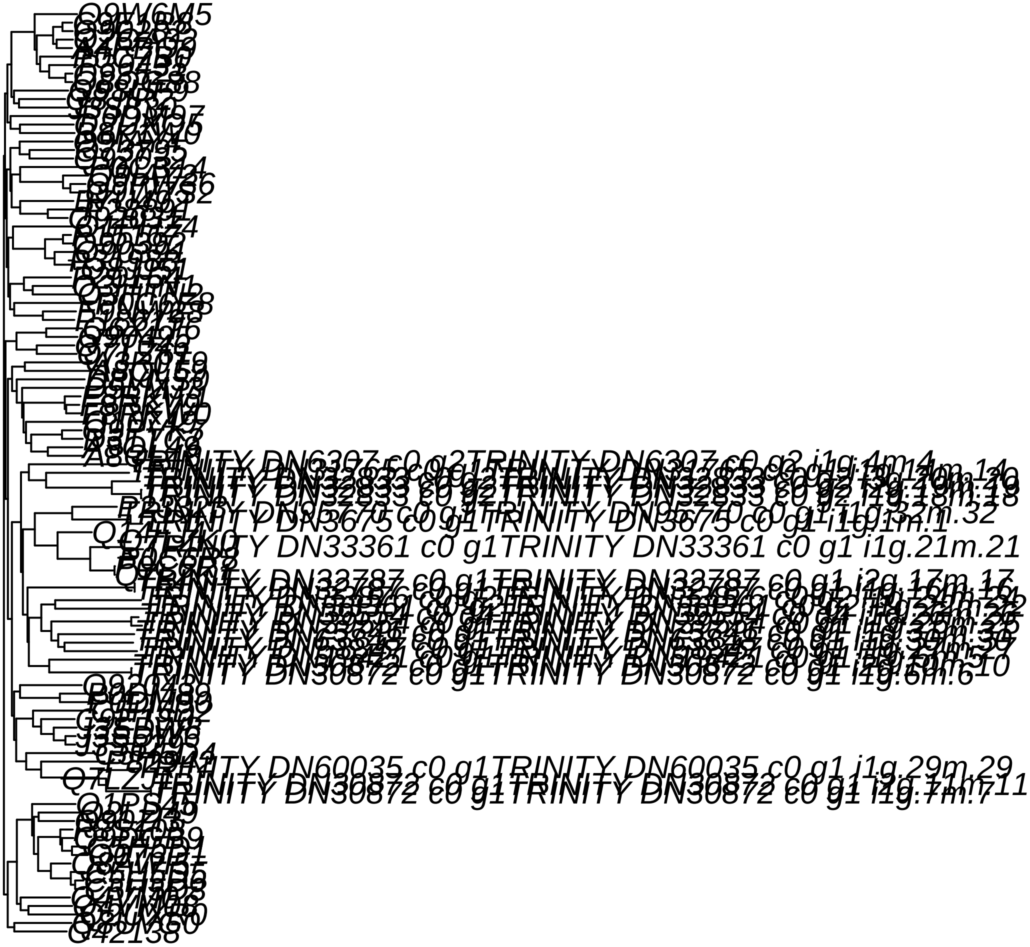
Q9W6M5
TRINITY DN6307 c0 g2TRINITY DN6307 c0 g2 i1g.4m.4
TRINITY DN3675 c0 g1TRINITY DN3675 c0 g1 i1g.1m.1
TRINITY DN33361 c0 g1TRINITY DN33361 c0 g1 i1g.21m.21
TRINITY DN32787 c0 g1TRINITY DN32787 c0 g1 i2g.17m.17
TRINITY DN30872 c0 g1TRINITY DN30872 c0 g1 i1g.6m.6
TRINITY DN60035 c0 g1TRINITY DN60035 c0 g1 i1g.29m.29
TRINITY DN30872 c0 g1TRINITY DN30872 c0 g1 i1g.7m.7
Q42138
